# CD33 (Siglec-3) Inhibitory Function: Role in the NKG2D/DAP10 Activating Pathway

**DOI:** 10.1155/2019/6032141

**Published:** 2019-04-15

**Authors:** Trinidad Hernández-Caselles, Rubén Corral-San Miguel, Antonio José Ruiz-Alcaraz, Pilar García-Peñarrubia

**Affiliations:** Departamento de Bioquímica, Biología Molecular B e Inmunología, Faculty of Medicine, IMIB-University of Murcia, Murcia, Spain

## Abstract

CD33 (siglec-3), a well-known target in leukemia therapy, is an inhibitory sialoadhesin expressed in human leukocytes of the myeloid lineage and some lymphoid subsets, including NK cells. It may constitute a control mechanism of the innate immune system; nevertheless, its role as an inhibitory receptor remains elusive. Using human NK cells as a cellular model, we analyzed CD33 inhibitory function upon different activating receptors. In high-cytotoxicity NKL cells, CD33 displayed a prominent inhibition on cytotoxicity triggered by the activating receptors NKG2D and, in a lower extent, 2B4, whereas it did not inhibit NKp46-induced cytotoxicity. NKp46 was partially inhibited by CD33 only when low-cytotoxicity NKL cells were tested. CD33 triggering did not inhibit IFN-*γ* secretion, contrasting with ILT-2 and CD94/NKG2A inhibitory receptors that inhibited cytotoxicity and IFN-*γ* secretion induced by all activating receptors tested. CD33-mediated inhibition of NKG2D-induced triggering involved Vav1 dephosphorylation. Our results support the role of CD33 as an inhibitory receptor preferentially regulating the NKG2D/DAP10 cytotoxic signaling pathway, which could be involved in self-tolerance and tumor and infected cell recognition.

## 1. Introduction

Siglecs (sialic acid-binding immunoglobulin-like lectins) are immunoglobulin-type transmembrane proteins that recognize sialylated ligands commonly found at terminal positions of cell surface glycoproteins and glycolipids. Most of the siglec family members are expressed on the surface of immune cells and function as inhibitory receptors since they possess ITIM and ITIM-like cytoplasmic motifs that, after engagement, become phosphorylated and transmit inhibitory signals by recruiting SHP-1 and/or SHP-2 phosphatases. Siglecs interact both in *cis* and in *trans* with their ligands which raises the possibility that siglecs inhibit cellular functions at both levels, self-membrane and after transmembrane interaction [1–3]. It has been hypothesized that siglecs maintain a constitutive inhibitory tone in their native state, as they were bound to sialo conjugates in *cis* when expressed on normal leukocytes [1, 2]. Increasing evidence indicates that inhibitory siglecs modulate inflammatory and immune responses through dampening of tyrosine kinase-driven signaling pathways as a mechanism of preventing self-reactivity [3].

CD33 (siglec-3) is the smallest siglec member. It preferentially binds to *α*2-6- and *α*2-3-sialylated glycans and strongly binds to sialylated ligands on leukemic cell lines [3–5]. Recently, the C1q complement system component has been established as a soluble ligand for CD33 [6]. CD33 is mainly expressed in the surface of human leukocytes of the myeloid lineage; however, it is important to note that it can also be expressed on lymphoid cells, including NK cells at several differentiation stages [7–12]. Previous works have clearly demonstrated that CD33 is working as an inhibitory receptor since, when crosslinked, it becomes phosphorylated on its cytoplasmic ITIM sequence that recruits SHP-1 and SHP-2 phosphatases and negatively regulates cell activation, in both myeloid cell lines and activated NK cells [4, 7, 13]. Human CD33 is expressed on the cell membrane as two isoforms, CD33M, the full-length protein, and CD33m, lacking the V extracellular domain [7]. The CD33 expression level has been recently related to Alzheimer's disease pathology [5], autoimmune diseases such as systemic lupus erythematosus (SLE) [6], type II diabetes [14], or infection [15]. However, CD33 inhibitory/regulatory function remains elusive. Expression of CD33 on activated T and NK cells provides new models to further study CD33 biological function.

NK cells are large granular lymphocytes of the innate immune system that provide protection against pathogens, tumor cells, and autoimmune responses. They represent the best-studied model in which a wide array of activating and inhibitory receptors regulates their cellular activity. Inhibitory receptor function is involved in self-tolerance maintenance in NK cells [16, 17]. CD16, NKp46, or NKp30 are the major triggering human NK receptors that, through association with different adaptor molecules, initiate different activation signaling cascades. Thus, CD16 associates with the CD3zeta and/or FcR*γ* ITAM- (immunoreceptor tyrosine-based activation motif-) bearing subunits which recruit ZAP70/Syk kinases [17]. NKG2D (associated with DAP10, a transmembrane adaptor molecule containing a YINM sequence) signals via recruitment of phosphatidylinositol 3-kinase (PI3K) and Grb2 (growth factor receptor-bound protein 2) [18, 19]. Other activating receptors such as 2B4 (CD244) or NTB-A (CD352) which contain TxYxxV/I cytoplasmic motifs permit association with the SLAM-associated protein (SAP) adaptor protein [20, 21]. The best known human inhibitory receptors (KIR, LILR, and lectin-like receptors such as CD94/NKG2A) are also expressed on NK cells and recognize mainly MHC class I molecules [16, 17].

In this work, we aimed to further explore the inhibitory role of CD33 and compare it with other well-known inhibitory receptors. To address this aim, we used NK cells that express CD33 and can be triggered through different activating and canonical inhibitory receptors. We found that CD33 modulated the function of non-ITAM-coupled NKG2D and 2B4 receptors and mediated effector/target adhesion through dephosphorylation of Vav1 signaling intermediary and cytoskeleton activation. Our data indicate that CD33, differently to canonical inhibitory receptors, may inhibit the function of some specific activating receptors, and thus, it could be involved in self-tolerance regulation.

## 2. Materials and Methods

### 2.1. Antibodies

The anti-CD33 mAb used were clone WM53 (IgG1, Serotec, Oxford, UK) which recognizes the Ig V domain on the CD33M isoform and clone HIM3-4 (IgG1, eBioscience, San Diego, CA) which recognizes the C_2_ domain on both CD33M and CD33m isoforms [8]. Other functional-grade purified mouse anti-human antibodies were as follows: anti-NKG2D clone 1D11 (IgG1) from eBioscience, anti-NKp46 (IgG2b) from RD Systems, anti-2B4 (C1.7, IgG1) from Immunotech (Marseille, France), and anti-NKG2A (Z199, IgG2a) from Beckman Coulter (Marseille, France). Isotype control Ab were from Sigma-Aldrich (Saint Louis, MI, USA). Fluorochrome-conjugated PE-WM53, FITC-anti-CD3, PE-anti-CD25, PE-anti-CD56, FITC-CD56, and PE-Cy5-anti-CD16 mAb were from BD Biosciences (Mountain View, CA, USA). PE-Cy5-HIM3-4 was from eBioscience. APC-Cy7-CD3, FITC-CD16, BV421-CD56, PE-Cy7-NKG2D, PE-Cy7-NKp46 (BioLegend), FITC-CD57 (BD Biosciences), and biotin-NKG2A (Miltenyi Biotec) were used for multicolor analysis of NK cells. KD1 (anti-CD16, IgG2a), HP3B1 (anti-CD94, IgG2b), HP-F1 (anti-ILT2, IgG1), Z199 (anti-NKG2A), HP-3E4 (anti-CD158a, IgM), and MAR93 (anti-CD25, IgG1) were kindly provided by Dr. A. Moretta (Milan, Italy) and Dr. M. López-Botet (Barcelona, Spain) and used as culture supernatants. W6/32 (anti-MHC class I, IgG2a) was obtained from ATCC.

Western blot primary antibodies were as follows: rabbit polyclonal anti-phospho-PI3K p85 (Tyr458)/p55 (Tyr199) Ab from Cell Signaling Technology; rabbit polyclonal anti-phospho-Vav1 (Tyr160) Ab from Invitrogen; rabbit polyclonal anti-CD33, rabbit polyclonal anti-SHP-2, and rabbit polyclonal anti-phospho-ERK1/2 (Thr202/Tyr204); rabbit anti-phospho-Akt/PKB (Ser473); and rabbit anti-phospho-p38 MAPK (Thr180/Tyr182), and their correspondent Ab against total proteins were from Santa Cruz Biotechnology® (Santa Cruz, CA, USA). Anti-Vav1 was from Sigma-Aldrich.

### 2.2. Effector and Target Cells and Immunophenotypic Analysis

The human NK cell line NKL (kindly provided by Dr. Michael J. Robertson, Indiana University (Bloomington, IN, USA)) was maintained (a) ranging from an exponential phase to plateau (approx. from 0.05 × 10^6^ cells/mL to 0.6 × 10^6^ cells/mL) or (b) growing under a continuous exponential growth phase (approx. from 0.025 × 10^6^ cells/mL to 0.25 × 10^6^ cells/mL) for two or more weeks, using complete tissue culture medium (MCC, composed of RPMI 1640, 10% heat-inactivated FBS, and antibiotics (PAA Laboratories, Pasching, Austria) and supplemented with 100 U/mL rIL-2 (Proleukin, Chiron, Amsterdam, Holland)). Additionally, NKL cells were grown for 24 h in the presence of 1000 U/mL rIL-2 before being used in the killing assays to increase their killer activity. The length of the exponential growth phase after the last plateau had important functional implications in NKL cells. Consequently, assays were mainly performed using 24-48 h exponentially growing NKL cells [22].

Polyclonal primary NK cells were obtained from blood samples of healthy volunteers. Collection of blood and all protocols were approved by the Ethics Committee of the University of Murcia and complied with the Declaration of Helsinki and the Good Clinical Practice guidelines. Volunteers gave written informed consent. Peripheral blood lymphocytes (PBL) were purified by discontinuous density gradient in LSM 1077 Lymphocyte separation medium (PAA Laboratories GmbH) according to standard protocols, and they were stimulated and expanded by coculture with irradiated allogeneic cells in the presence of IL-2 (100 U/mL) as described [7]. After 6-day stimulation, cells were maintained in MCC supplemented with 100 U/mL of rIL-2 until quiescence (low expression of surface CD25 antigen) which occurred approximately three-four weeks after stimulation. Then, quiescent primary NK cells were negatively selected using anti-CD3 (OKT3) and goat anti-mouse-coated magnetic beads (Dynabeads, Invitrogen, Oslo, Norway). The resulting populations were shown to be ≥95% CD3^─^CD16^+^CD56^+^. Phenotypic analysis of cells was carried out by flow cytometry using a FACScan and a LSRFortessa X-20 cytometer (Becton Dickinson). CellQuest software (Becton Dickinson) and Flowing 2.5.1 (http://flowingsoftware.btk.fi) software were used [7].

NK target cells were the mouse mastocytoma FcR^+^ P815 (ATCC), the MHC-I-deficient erythroblastoid cell line K562, and the EBV-transformed lymphoblastoid cell line 721.221.

### 2.3. Redirected Cytotoxicity Assays

The redirected lysis assay was performed as previously described [22] coculturing NKL cells with ^51^Cr-labeled P815 target cells and optimized concentrations of the following soluble mAb against activating and/or inhibitory receptors or control Ig: anti-CD16 (1/40 diluted culture supernatant), anti-NKp46 (0.34 *μ*g/mL), NKG2D (0.34 *μ*g/mL), 2B4 (0.17 *μ*g/mL), anti-MHC-I (1/40 diluted culture supernatant), anti-CD33 (WM53 or HIM3-4, 1.25 *μ*g/mL), anti-ILT2, anti-NKG2A or anti-CD94 (1/20 diluted culture supernatants), and control Ig (1.25 *μ*g/mL). Isotype control antibodies IgG1 and IgG2a were included in all inhibitory experiments alone or together with antibodies against activating receptors when anti-CD33 or anti-NKG2A mAb were tested, showing no effect. IgG2b was previously tested showing also no effect on cytolytic assays [22].

NKL apoptosis was assessed determining the percentage of CD25^+^ annexin V^+^ NKL cells by flow cytometry after the 4 h killing assay. The inhibitory effect of CD33 away from activating receptors was tested by performing the redirected lysis assay using 96-well ELISA plates (Nunc, Roskilde, Denmark) coated with anti-CD33 mAb or isotype control immunoglobulins.

Spontaneous killing of P815 cells by NKL cells was very low (3.6 ± 2.7% lysis at a 20 : 1 E/T ratio) indicating that, in our experimental setting, the interaction of NKL activating receptors with putative murine ligand(s) [22] was not significant.

### 2.4. Cytokine Production Assays

We studied IFN-*γ* production by NKL (25000 cells/well, grown in 100 U/mL rIL-2) cocultured with P815 cells at a 1 : 1 E/T ratio in the presence of the same combination of mAb but using twice the Ab concentration. After 22-24 h incubation, cell-free supernatants were collected and tested for IFN-*γ* concentrations by ELISA (eBioscience) [22].

### 2.5. Quantitation of Percentages of Inhibition on Cytotoxicity and Cytokine Production

Results reported as the percentage of inhibition were calculated as follows:

(1) $$\begin{array}{c} \%\text{ inhibition of cytotoxic function}=100-\frac{\%\;{\text{lysis}}_{\text{Activ}.+\text{Inhib}.}-\%\;{\text{lysis}}_{\text{isotype control}}}{\%\;{\text{lysis}}_{\text{Activ}.+\text{isotype control}}-\%\;{\text{lysis}}_{\text{isotype control}}}\times 100, \end{array}$$

(2) $$\begin{array}{c} \%\text{ inhibition of IFN}‐\gamma \text{ secretion}=100–\frac{{\text{IFN}}_{\text{Activ}.+\text{Inhib}.}-{\text{IFN}}_{\text{isotype control}}}{{\text{IFN}}_{\text{Activ}.+\text{isotype control}}-{\text{IFN}}_{\text{isotype control}}}\times 100. \end{array}$$

### 2.6. Sialidase and Periodate/Borohydride Treatments

Sialidase treatment or mild periodate/borohydride oxidation/reduction was used to eliminate homotypic or heterotypic CD33 *cis* interactions as described [8, 23]. Briefly, NK cells were treated with *Arthrobacter ureafaciens* sialidase (Roche, Mannheim, Germany) at 10-20 mU/10^6^ cells in RPMI 1640 pH 6.9 for 1.5 h at 37°C and then extensively washed with 2% FCS in PBS. For mild periodate oxidation, cells (1 × 10^6^ cells/mL) were incubated with sodium periodate (1 mM, Sigma-Aldrich) in PBS for 30 min on ice and then quenched with 1 mM glycerol and washed twice with cold PBS. Cells were then treated with freshly prepared sodium borohydride (20 mM) for 10 min to reduce the aldehydes generated by periodate. They were washed again with PBS and culture medium. Finally, cells were counted and used for functional assays or stained with the corresponding mAb or with biotinylated Sambucus nigra lectin (SNA, Vector Laboratories, Burlingame, CA, USA) to test the effectiveness of the treatment. Cell viability after treatment was determined by using the MTT assay [24].

### 2.7. Receptor Crosslinking and Western Blotting Analysis

To evaluate the intracellular phosphorylation pattern, NKL cells were starved for 4 h in RPMI 1640 medium supplemented with 1% FBS and then incubated with primary antibodies (1 *μ*g/10^6^ cells) against CD33, NKG2A inhibitory receptors, or control MHC-I molecules plus anti-CD16, anti-NKG2D, anti-NKp46, or anti-2B4 mAb on ice. Cells were then crosslinked with sheep anti-mouse F(ab′)_2_ Ab and stimulated for 5-10 min at 37°C. Stimulation was stopped on ice, and then cells were pelleted, lysed (lysis buffer from Cell Signaling Technology (Danvers, MA, USA) supplemented with 1 mM PMSF), and analyzed by Western blot as described [8]. Immunoblots were detected by using the Enhanced Chemiluminescence System© (ECL 2 Western Blotting Substrate, Thermo Scientific, Rockford, IL, USA). Protein bands were quantified by densitometry using Aida software and expressed as relative to total protein.

### 2.8. CD33/SHP-2 Coprecipitation

Exponentially growing NKL or purified activated primary NK cells were lysed at a concentration of 10^7^ cells/mL, and then 350 *μ*L lysates were incubated with 1 *μ*g WM53, Z199, or IgG1 isotype control Ab and protein G-coated magnetic microbeads (Miltenyi Biotec GmbH, Germany) for 1 h on ice. Separation was performed following the manufacturer's protocol.

### 2.9. Statistical Analysis

Data are reported as mean ± standard deviation or mean ± SEM. Statistical differences were analyzed using Student's *t*-test. Calculations were performed using the SPSS 21.0 software (Chicago, IL, USA).

## 3. Results

### 3.1. Functional Characteristics of NKL Cells under Different Culture Conditions

**Figure 1:**
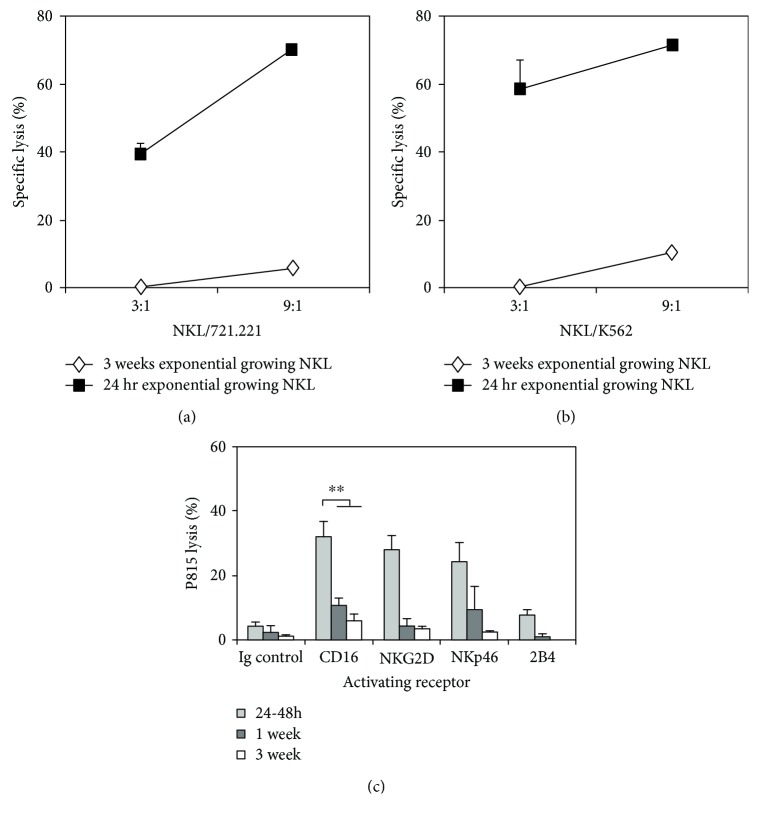
Functional differences of NKL cells cultured at different growing conditions. Cytotoxic ability against (a) 721.221 or (b) K562 target cells displayed by 24 h (■) or 3 weeks (◊) exponentially growing NKL cells, as described in Materials and Methods. One representative experiment out of three with similar results is shown. (c) Redirected cytotoxicity against P815 cells in the presence of mAb against CD16, NKp46, NKG2D, or 2B4 activating receptors by NKL cells (3 : 1 E/T ratio) grown at an exponential phase for 24 h, 1 week, or 3 weeks. According to these data, the 24-48 h exponentially growing NKL cells were referred as high-cytotoxicity NKL cells (hc-NKL). Data show the mean ± SEM of P815 lysis percentages of a minimum of three independent experiments. All differences are statistically significant. The highest cytotoxic ability was displayed by 24 h exponentially growing NKL cells. ^∗∗^ *P* < 0.01.

First, we explored the suitability of NKL cells as a model to study the CD33 inhibitory receptor upon the activity triggered by distinct killer activating receptors (KARs) since these cells constitutively express CD33 (detected by both WM53 and HIM3-4 anti-CD33 mAb) as well as several activating (CD16, NKp46, NKG2D, and 2B4) and inhibitory (ILT2 and CD94/NKG2A) receptors (Supplementary Figure S1A). NKL cells did not express KIR2DL1, 2DS1, 2DL2, 2DL3, 2DS2, or 3DL1 receptors (not shown). We found that NKL cells, grown following exponential/steady-state three-day cycles, compared to cells growing constantly at an exponential rate, did not show changes in the expression of CD33 or other studied receptors (not shown). However, NKL cytotoxic activity was dependent on the cell growing conditions, displaying a high cytotoxic activity against both 721.221 and K562 target cells when growing at the exponential phase for a short period of time (24-48 h) and a very low cytotoxic capability against the same targets when growing at the long-term exponential phase (Figures 1(a) and 1(b)). Similar to natural cytotoxicity, the redirected killing activity of NKL cells, triggered by several KAR against P815, decreased as the length of the NKL exponential growth phase increased (Figure 1(c)). Long-term exponentially growing NKL cells recovered their cytotoxic capability when culture conditions were readapted (data not shown). Thus, we selected NKL cells growing under the exponential phase for 24-48 h as the optimum time in which these effector cells displayed their highest cytotoxic capability. They are referred to as high-cytotoxicity NKL (hc-NKL) cells.

### 3.2. CD33 Modulation of the Cytolytic Activity Triggered by Different Activating Receptors on High-Cytotoxicity NKL Cells

**Figure 2:**
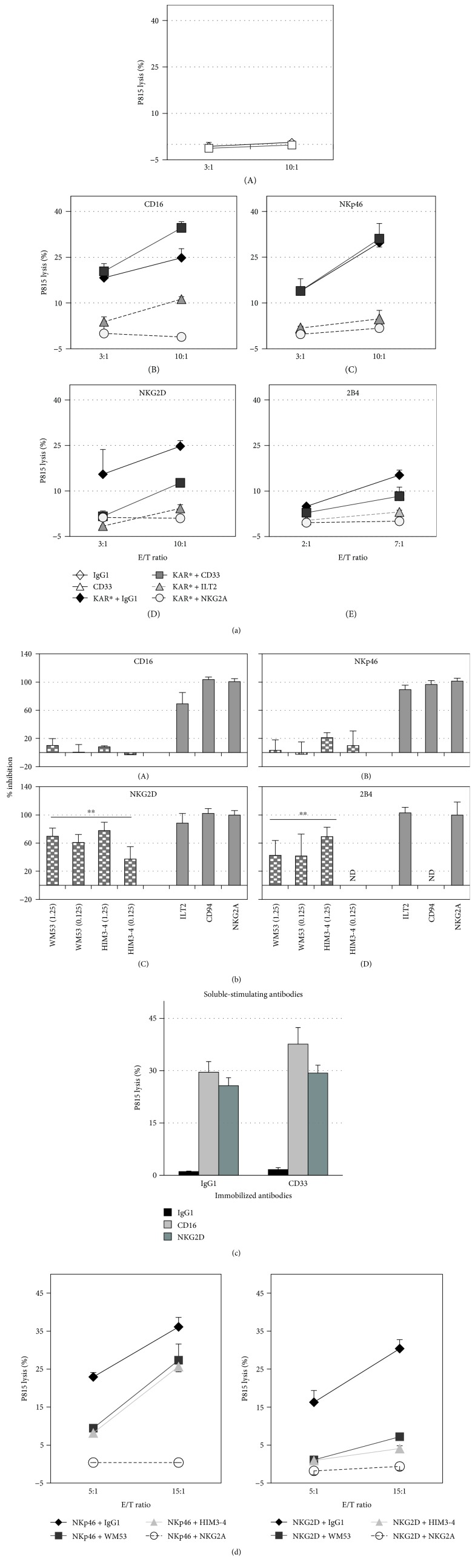
CD33 engagement inhibits cytotoxicity on hc-NKL cells. (a, b) hc-NKL cells were cocultured with ^51^Cr-P815 cells plus mAb against killer activating receptors (KAR^∗^): CD16 (a), B and (b), A, NKp46 (a), C and (b), B, NKG2D (a), D and (b), C, 2B4 (a), E and (b), D; control Ig or CD33 (WM53), ILT2, or the NKG2A inhibitory receptor in the ^51^Cr release assay. We show one representative assay out of five. (b) Percentages of inhibition (mean ± SD) obtained in all assays performed at all E/T ratios used. CD33 mAb are WM53 and HIM3-4. Numbers in parentheses are concentration in *μ*g/mL. N.D.: not determined. (c) CD33 ligation away from activating receptors by using immobilized anti-CD33 mAb. The figure shows the mean ± SD from two assays performed. Differences are statistically significant versus controls (without inhibitor or with constitutive inhibitors). ^∗∗^ *P* < 0.01. (d) CD33 engagement may partially inhibit cytotoxicity triggered by NKp46 on one-week exponentially growing low-cytotoxicity NKL cells. One representative assay is shown out of three with similar results.

Next, we examined the inhibitory role of CD33 using hc-NKL cells and the antibody-redirected killing assay against FcR^+^ P815 cells, since pairs of activating and inhibitory receptors can be crosslinked [25]. We found that hc-NKL cells did not significantly kill P815 cells when triggered with anti-CD33, anti-ILT2, anti-CD94, anti-NKG2A, or irrelevant mAb alone (Figure 2(a), A) and killed them when mAb against CD16, NKp46, NKG2D, or 2B4 activating receptors were used alone or coligated with an isotype control mAb. As expected, P815 killing was inhibited when mAb against CD16, NKp46, NKG2D, or 2B4 were colligated with mAb specific for canonical ILT2 or CD94/NKG2A inhibitory receptors. However, anti-CD33 mAb strongly reduced P815 killing only when triggered by NKG2D (69.6 ± 11.6% reduction, WM53 mAb at 1.25 *μ*g/mL, and a E/T ratio of 3 : 1 to 20 : 1) or 2B4 (41.5 ± 21.8% WM53 at 1.25 *μ*g/mL, and a E/T ratio 3 : 1 to 9 : 1) activating receptors (Figures 2(a) and 2(b)). Anti-CD16 or anti-NKp46-induced cytotoxicity was only slightly inhibited by several concentrations of anti-CD33 mAb, i.e., WM53 mAb decreased the lysis by 10.0 ± 9.5% and 2.8 ± 14.9% and HIM3-4 mAb produced a 7.8 ± 1.8% and 20.6 ± 7.0% of inhibition at 1.25 *μ*g/mL and 0.125 *μ*g/mL, respectively. Occasionally, WM53 or HIM3-4 produced a slight increase in the lysis induced by anti-CD16 or NKp46 mAb (Figures 2(a) and 2(b)). CD33-induced inhibition was not due to the induction of apoptosis in NKL cells (data not shown and [7]). Percentages of inhibition were dependent on the Ab-relative concentration used to crosslink both inhibitory and activating receptors (Figure 2 and data not shown). The lower CD33 inhibition exerted upon CD16 or NKp46 stimulation was not due to an insufficient concentration of inhibitory mAb since the assayed concentration of WM53 mAb almost saturated CD33 surface molecules on NKL cells as determined by flow cytometry (data not shown). Furthermore, the same amounts of WM53 mAb indeed inhibited, in parallel assays, the killing induced by NKG2D or 2B4 activating receptors. The use of a tenfold dilution of anti-CD33 mAb still displayed a significant inhibitory effect (Figure 2(b)). CD33 sequestering, using immobilized anti-CD33 mAb, could not inhibit P815 killing induced by anti-CD16 or anti-NKG2D soluble stimulators indicating the specific inhibitory effect of CD33 when co-crosslinked with NKG2D (Figure 2(c)).

The possibility of an artefactual inhibition caused by competition between activating and inhibitory mAb for binding to P815 FcR was explored by carrying out NKG2D-mediated cytotoxicity when noninhibitory receptors such as CD25 or HLA-E (with comparable expression to that of CD33 on NKL) were coengaged, in parallel with the corresponding dose response experiments with WM53 Ab. Results demonstrated that similar concentrations of both anti-CD25 and anti-HLA-E did not reduce cytotoxicity induced by NKG2D alone. On the contrary, WM53 mAb were able to inhibit the lysis mediated by anti-NKG2D (0.25 *μ*g/mL) at concentrations as low as 0.075 *μ*g/mL (Supplementary Figure S2). Crosslinking of other surface receptors, including adhesion molecules (CD2, CD58, ICAM-1, ICAM-3, CD29, or CD44), with NKG2D, NKp46, or CD16 cytolytic receptors did not significantly decrease or increase cytotoxicity against P815 cells as previously shown by us [22]. NKL/P815 conjugation experiments (Supplementary Table S1) demonstrated that coengagement of CD33 with the activating receptors CD16, NKG2D, and 2B4, increased conjugation percentages in all cases. In summary, all these results indicate that under our experimental conditions, there was no significant competition for P815 FcR.

The inhibitory effect of CD33 molecules was also tested on low-cytotoxicity NKL cells (long-term exponentially growing cells) triggered by NKp46 or NKG2D. Our results indicated that both anti-CD33 mAb WM53 or HIM3-4 negatively modulated P815 killing triggered by these activating receptors (Figure 2(d)). CD33 modulation of CD16-induced cytotoxicity against P815 cells (not shown) was consistently observed when we used low-cytotoxicity NKL cells as it was shown in our previous work [7].

Altogether, our results clearly show that CD33 is an inhibitory receptor that preferentially modulates cytolysis triggered by NKG2D or 2B4, both non-ITAM-bearing receptors, when NKL cells display their maximal cytolytic activity. Our results also showed that CD33 downregulates cytotoxic activity triggered by NKp46 or CD16 ITAM-containing receptors only when the NKL cytotoxic capability is low. NKG2D-induced cytotoxicity could only be modulated when both NKG2D and CD33 molecules were co-crosslinked.

### 3.3. IFN-*γ* Secretion Is Poorly Modulated by the CD33 Receptor

**Figure 3:**
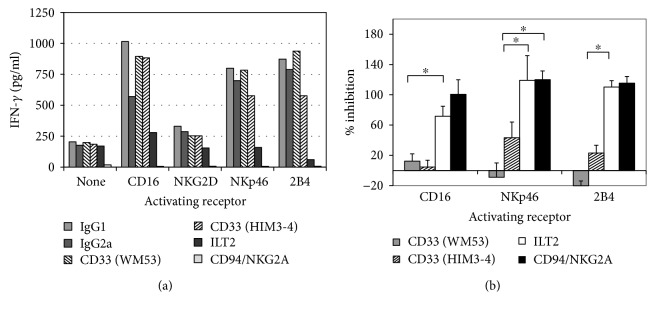
Inhibition of IFN-*γ* secretion by CD33 on NKL cells. hc-NKL cells were cocultured with P815 cells at a 1 : 1 E/T ratio. IFN-*γ* secretion is efficiently inhibited by anti-ILT2 but not by anti-CD33 (WM53 or HIM3-4) mAb. The figure shows (a) IFN-*γ* secretion (one representative assay out of four) and (b) percentage of inhibition (mean ± SEM) of anti-CD33 and anti-ILT2 mAb from all assays performed. All differences are statistically significant. ^∗^ *P* < 0.05.

Next, we evaluated the inhibitory role of CD33 upon IFN-*γ* secretion triggered in hc-NKL cells (Figures 3(a) and 3(b)). We found no significant increase of IFN-*γ* production when cells were triggered by anti-CD33 (WM53 or HIM3-4), anti-NKG2A, anti-ILT2, or irrelevant mAb alone. In agreement with reported data [26], IFN-*γ* secretion was weakly triggered by anti-NKG2D mAb but it was strongly induced when triggered through CD16, NKp46, or 2B4 (Figure 3(a)). Coligation with ILT2 or NKG2A dramatically inhibited IFN-*γ* secretion (71.8 ± 21.7% to 110.3 ± 17.8% of inhibition; Figures 3(a) and 3(b)). In contrast, CD33 (WM53) had no effect although occasionally, the HIM3-4 anti-CD33 mAb could partially inhibit the IFN-*γ* secretion when triggered by NKp46 and 2B4 activating receptors (43.3 ± 17.4% and 23.1 ± 24.1% inhibition, respectively). Altogether, our results showed that CD33 lacks significant inhibitory effect on cytokine production by hc-NK cells, whereas canonical inhibitory receptors such as ILT2 and CD94/NKG2A are able to neutralize almost completely cytokine secretion.

### 3.4. CD33 Expression and Function on Primary Human NK Cells

**Figure 4:**
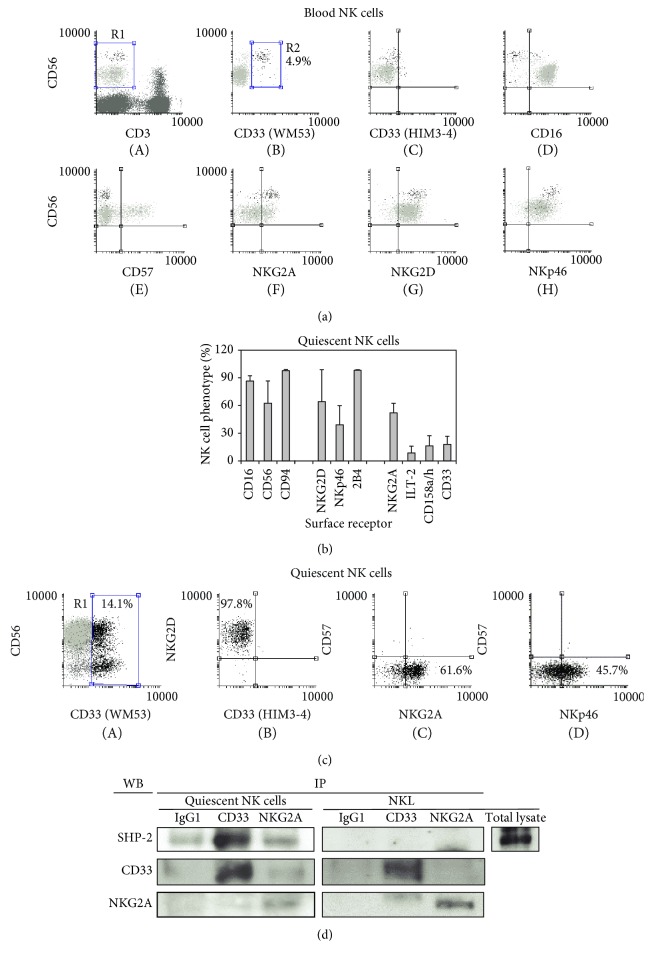
CD33 expression and function in primary human NK cells. (a) Blood NK cells (CD3^−^CD56^+^ lymphocytes in region R1) and (b, c) quiescent primary human NK cells were phenotyped by flow cytometry. (a) Blood CD33^+^ NK cells were mainly CD56^bright^ (R2 region, dark dots), CD16^−^, CD57^−^, NKG2A^+^, and NKG2D^+^. The figure shows a representative blood sample out of the four tested. (b) Purified (95%) quiescent NK cells were mainly CD56^bright^CD16^+^ and show partial expression of NKp46, NKG2A, ILT-2, CD158a, and CD33 antigens. The figure shows percentages of NK cells expressing different surface receptors among all NK cell cultures obtained from seven healthy individuals. (c) CD33^+^ (WM53^+^ HIM3-4^−^) quiescent NK cells (R1 region, dark dots) were CD57^−^, NKG2D^+^, and partially expressed NKp46 and NKG2A antigens. (d) CD33 coprecipitated SHP-2 phosphatase only in quiescent primary human NK cell lysates. The figure shows a representative experiment out of two with similar results.

We examined CD33 expression on healthy donors' blood lymphoid cells and found a CD3^−^CD56^bright^CD16^−^ WM53^+^ HIM3-4^−^ small population (0.52 ± 0.40%; *n* = 9) of NK cells (Figure 4(a)), phenotypically similar to the CD56^+^CD33^+^ NK cell subpopulation previously described by Handgretinger et al. in human umbilical cord blood [9]. Further analysis revealed that WM53^+^ NK cells were CD57^−^ and NKG2A^+^ and expressed the highest levels of NKG2D and NKp46 (Figure 4(a), E–H).

Quiescent NK cell populations, obtained after stimulation, showed high percentages of CD56^+^, CD16^+^, 2B4^+^, and CD94^+^ cells. Percentages of NKG2D^+^ and NKp46^+^ cells were variable among individual cultures, whereas low percentages of ILT-2^+^ and CD158a^+^ cell populations were found. CD33^+^ (WM53^+^ HIM3-4^−^) quiescent NK cells represented 17.9 ± 8.7% among NK cell cultures, ranging from 5% to 35% (Figure 4(b)). CD33^+^ NK cells were CD57^−^ and NKG2D^+^, showing variable expression of NKp46 and NKG2A surface receptors (Figure 4(c)).

The enriched NK populations presenting the highest number (23-35%) of CD33^+^ NK cells were then tested for redirected killing activity against P815 cells. Results showed that, contrarily to NKL cells, NKG2D- or 2B4-mediated lytic activity of CD33^+^ NK cells was not inhibited by WM53 anti-CD33 mAb (Supplementary Figure S3). Since HIM3-4 anti-CD33 mAb do not recognize CD33 molecules on primary NK cells, the above results suggest a regulatory mechanism of CD33 function in primary NK cells that could be related to molecular modification of CD33 itself, that is, glycosylation and/or association with other membrane proteins as previously reported on both lymphoid and myeloid cells [6–8].

To further explore CD33 function in primary human NK cells, we removed cell surface sialic acid by neuraminidase [8] or by mild periodate/borohydride (PER/BOR) treatment [23]. Neuraminidase-treated NK cells were stained by both WM53 and HIM3-4 anti-CD33 mAb (not shown), as we previously described for T lymphocytes [8]. In turn, PER/BOR treatment reduced the binding of anti-CD33 mAb. Unfortunately, both treatments notably diminished or completely abolished cytotoxicity triggered by all activating receptors in primary NK cells (not shown), as previously shown for neuraminidase treatment [27], which hampered this part of our work.

### 3.5. SHP-2 Phosphatase Coprecipitates with CD33 in Human Primary NK Cells

To further explain the lack of inhibitory capacity of CD33 in primary human NK cells, we tested the presence of SHP-2 basally associated with CD33 as an indicator of a presumable inhibitory state of this siglec. We found that, differently to control IgG1 or NKG2A, CD33 and SHP-2 phosphatase coprecipitated from purified quiescent NK cells (Figure 4(d)). CD33 and NKG2A did not coprecipitate with SHP-2 from hc-NKL cells. Thus, CD33/SHP-2 phosphatase association together with the null staining with HIM3-4 anti-CD33 mAb strongly suggests a likely CD33 association in *cis* with cell membrane ligands in agreement with a previous report [6]. Our results support the hypothesis that CD33 can modulate cytotoxicity when crosslinked with NKG2D and uncoupled with *cis* ligands/intracellular proteins such as SHP-2 phosphatase, as shown on NKL cells.

### 3.6. CD33 Inhibition of Killing Triggered by NKG2D Is Mediated by Counteracting Vav1 Phosphorylation Levels

**Figure 5:**
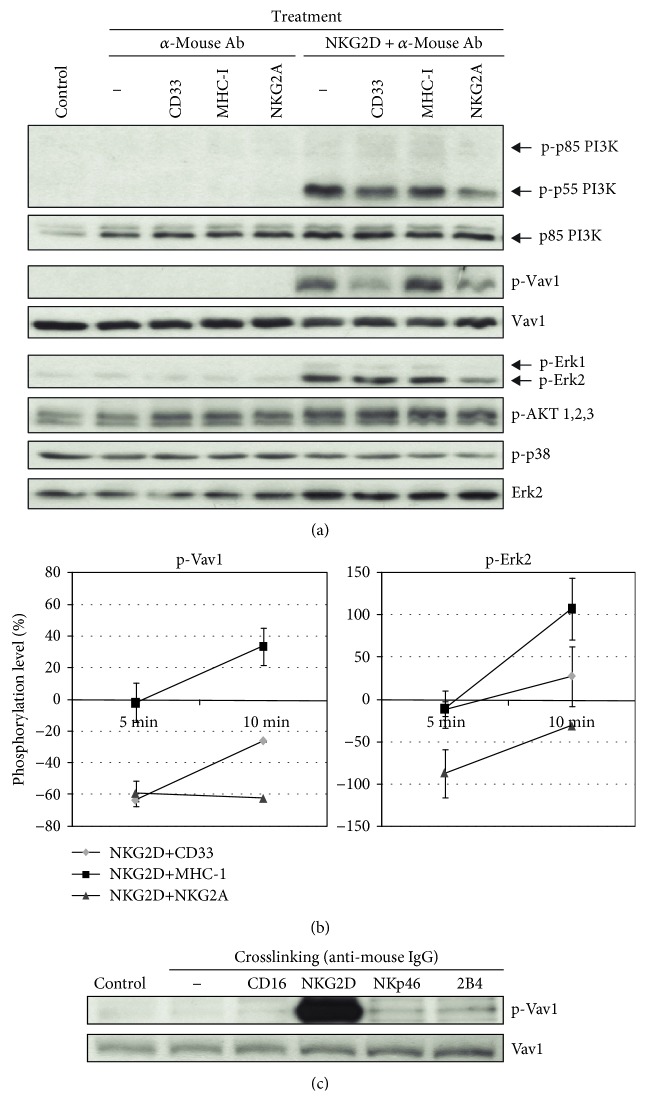
CD33 ligation reduces Vav1 phosphorylation under crosslinking with NKG2D activating receptor in hc-NKL cells. (a) hc-NKL cells were treated with the indicated combination of antibodies against NKG2D and/or CD33, NKG2A, or MHC-I as a control, crosslinked with sheep anti-mouse Ab on ice and incubated at 37°C for 5 min. Then cellular extracts were analyzed by Western blotting to test the indicated phosphorylated intracellular proteins. Crosslinking of NKG2D plus CD33 resulted in a reduction of the amount of p-Vav1 whereas crosslinking of NKG2D plus NKG2A resulted in a reduction of both p-Vav1 and p-ERK2 intracellular mediators. Data are representative of four independent experiments. (b) Changes in p-Vav1 and p-ERK2 over time are represented as percentage of phosphorylated proteins after NKG2D plus CD33, MHC-I, or NKG2A stimulation in relation to NKG2D stimulation alone. Means and SDs from four experiments are shown. (c) Relative extent of Vav1 phosphorylation in hc-NKL cells when stimulated by crosslinking of CD16, NKG2D, NKp46, or 2B4 activating receptors.

To further examine the mechanism involved in CD33-mediated inhibition of NKG2D signaling cascade, we explored the intracellular phosphorylation status of several signal transduction molecules triggered by NKG2D using hc-NKL cells. Hence, phosphorylation levels of p55 and p85 PI3K subunits, Vav1, ERK2, p38, or AKT kinases were determined by Western blot analysis at 5 and 10 minutes after crosslinking pairs of receptors with an anti-mouse F(ab′)_2_ Ab, using MHC-1 and NKG2A as negative and positive control molecules, respectively (Figure 5). As expected, crosslinking of NKG2D alone or coligated with control MHC-1 molecules induced a rapid phosphorylation of the p55 PI3K subunit, revealing PI3K pathway activation, albeit the phosphorylated subunit p85 was not detectable in our blots (Figure 5(a)). NKG2D and NKG2A coengagement produced a sustained reduction of phospho-Vav1 (p-Vav1) and phospho-ERK2 (p-ERK2) both at 5 and 10 min stimulation. NKG2D coligation with CD33 notably reduced p-Vav1 levels (25-60% reduction of the maximum Vav1 phosphorylation; Figure 5(b)), whereas the p-ERK2 level remained unchanged. There was no significant modification on p38 MAP kinase or AKT phosphorylation at any experimental condition. We conclude that CD33 modulates signaling pathways in NKL cells through dephosphorylation of the important PI3K/Vav1 pathway like other canonical inhibitory receptors such as NKG2A. However, CD33 was not able to inhibit the MAP kinase pathway which is responsible for IFN-*γ* production [28]. These results were consistent with the corresponding functional assays described above and established a direct relationship between the CD33 ability to inhibit cytotoxic function and the decrease in Vav1 phosphorylation.

We also studied the p-Vav1 level after CD16, NKp46, or 2B4 stimulation in hc-NKL cells at several time points. In this regard, CD16, NKp46, or 2B4 stimulation induced very low and sometimes undetectable levels of p-Vav1 compared to NKG2D stimulation in our NKL cells (data not shown and Figure 5(c)). Since Tyr160 is one of the key regulatory sites that mediates Vav1 phosphorylation-dependent activation in the cell signaling process [29], our results suggest that the CD33-mediated inhibition of cytotoxicity works by impairing the Vav1 downstream pathway. Moreover, canonical inhibitory receptors such as CD94/NKG2A, besides acting on the PI3K/Vav1 pathway, may also exert their inhibitory control on distinct intracellular intermediates, since they could completely inhibit cytotoxicity when NKL cells were stimulated through ITAM-bearing activating receptors such as CD16 or NKp46 that produced low p-Vav1 levels, as shown here. We suggest that CD33 inhibition may rely at least in part on the ability to decrease Vav1 phosphorylation.

## 4. Discussion

The control of immune cells is determined by a fine balance between signals triggered from cell surface activating and inhibitory receptors. The contribution of each activating/inhibitory pair is difficult to assess because multiple receptor-ligand interactions occur during natural processes. The precise inhibitory functions of CD33 still remain unclear. Herein, we used the NKL/FcR^+^ P815 crosslinking model which allows to engage pairwise comparison of selected activating and inhibitory receptors since (a) the FcR^+^ P815 cell line was scarcely recognized by NKL cells, (b) the natural cellular ligands of CD33 are unknown, and (c) this model provides an appropriate tool to study inhibitory receptor features, as previously shown [22].

Our results provide evidence that CD33 can exert an inhibitory function when crosslinked with specific activating receptors. Thus, in hc-NKL cells, CD33 acted as a unique receptor that efficiently and specifically antagonized the cytotoxic response mediated by the DAP10-coupled specific activating receptor NKG2D or the SAP-associated 2B4. Contrarily, CD33 hardly inhibited IFN-*γ* production. Moreover, CD33 partially modulated cytotoxicity triggered by receptors coupled to ITAM containing subunits such as CD16 or NKp46 only when the cytotoxic capability of NK cells was reduced. Compared to canonical inhibitory receptors ILT2 or CD94/NKG2A, which strongly regulate both cytotoxicity and cytokine production triggered by all activating receptors, CD33 displays a fine-tune, more restricted inhibitory function.

We show that CD33 is expressed on a subset of blood CD56^bright^CD16^−^ NK cells, a similar NK cell subset previously found in human umbilical cord blood [9]. Additionally, CD56^bright^CD16^−^ NK cells have been found among the NK subsets that predominate in several tissues such as the decidua, liver, gastrointestinal mucosa, or skin (reviewed by Sharma and Das [30]), although CD33 expression was not determined in the above cases. NK CD56^bright^CD16^−^ NK cells have been found *in vivo* at increased proportions during IL-2 therapy [31], in affected tissues by pathological inflammatory conditions like rheumatoid arthritis [32] and ex vivo IL-2-expanded primary NK cells [12]. Our *in vitro* activated NK cells were mainly CD56^bright^CD16^+^, although CD33 was expressed on both CD56^bright^ and CD56^−^ subsets. Functionally, these NK cells were highly responsive cells (high levels of target killing were found at low E/T ratios) and phenotypically like the activated NK cells generated by other researchers [12, 30, 33, 34] with a potential use in cell-based therapy against human malignancies [34, 35]. Up to date, the significance of these phenotypes remains unknown.

Siglecs expressed on normal cells are “masked” (probably bound to ligands in *cis*) when they do not bind to *trans* ligands in *in vitro* assays [1, 2, 36]. Since their cellular ligands are unknown, sialic acid depletion by using neuraminidase or periodate treatment is the only available method to address this issue. However, these methods may be deleterious to the cells and/or to the membrane receptors ([27] and this work) preventing functional studies. The occurrence, mechanism, and biological relevance of the masking phenomenon require further investigation. In this work and previous works, WM53^+^ lymphoid and myeloid cells were undetected or poorly detected by HIM3-4 mAb (that recognizes the C_2_-Ig-like domains) [6, 8]. Immunoprecipitated CD33 was constitutively associated with SHP-2 phosphatase (Figure 4). These features suggest a probable CD33-homotypic or heterotypic association with other membrane molecules on the surface of primary NK cells, since sialidase treatment increased HIM3-4 staining of activated human NK cells. Thus, the cytotoxic activity of NK cell populations could be at least in part modulated by a siglec-mediated inhibitory mechanism likely determined by its *cis* or *trans* molecular interactions. In this sense, it is well known that peripheral blood CD56^bright^ NK cells display lower natural cytotoxic activity than their counterpart CD56^dim^ cells [37]. Besides, cord blood-derived CD33^+^ NK cells display lower cytolytic activity against target cells than their CD33^–^ counterparts [9]. New tools allowing further *in vitro* manipulation of the *cis*/*trans* ligand interaction state (transition) of siglecs are needed in order to accomplish this issue.

**Figure 6:**
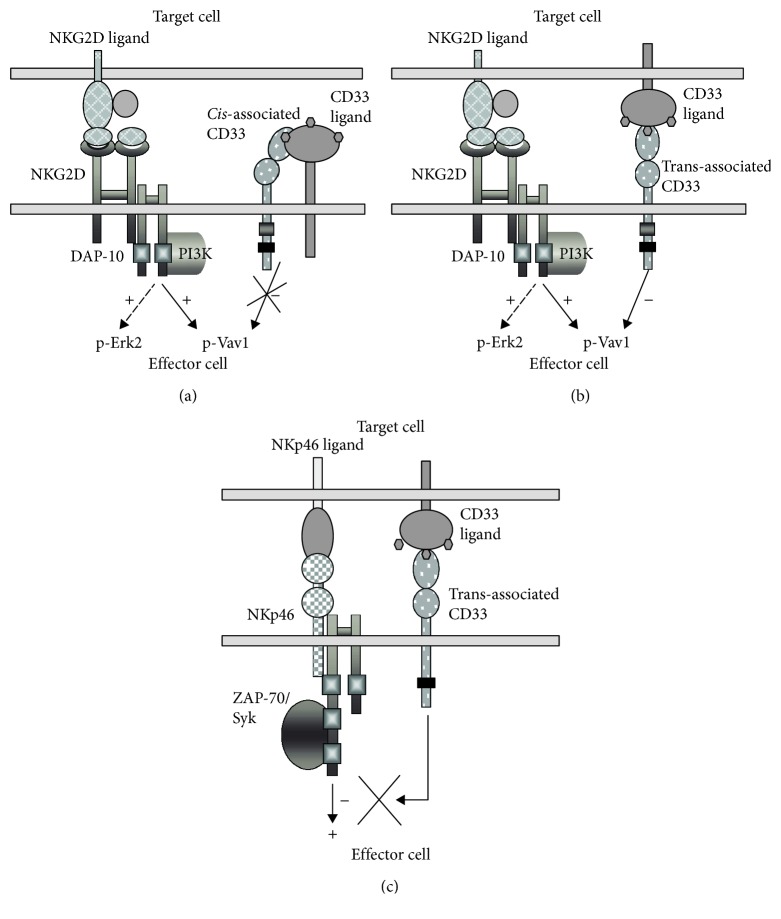
Model for CD33 inhibition in NK cells. *Trans*-associated (b) but not *cis*-associated (a) CD33 receptors may be inhibitory receptors for effector cells activated by NKG2D. (c) CD33 receptors may not be inhibitory for ITAM-bearing killing activating receptors such as NKp46. It is proposed that Vav1 is a critical intermediary in CD33 inhibitory effect on NKG2D-mediated cytotoxicity.

Regarding the inhibitory role, our results show that CD33 engagement interferes with Vav1 and probably p55/PI3K phosphorylation triggered by NKG2D crosslinking as described in Figure 6. Vav1 regulates the actin cytoskeleton and degranulation of cytotoxic cells; it has been identified as a direct substrate of SHP-1 in NK cells [38] and a central signaling molecule in determining the cytotoxic activity of NK cells [39]. Importantly, mice lacking Vav1 have diminished cytolytic activity against several tumor targets but retain the ability to produce cytokines [40]. In our model, the lack of CD33 inhibitory activity upon CD16 and NKp46 crosslinking is associated with low levels of Vav1 phosphorylation (Figure 5(c) and 6). At the same time, CD33 engagement could not interfere with ERK2 phosphorylation and cytokine production, while NKG2A highly reduced Vav1, p55 PI3K, ERK2 phosphorylation, and IFN-*γ* secretion. In accordance with previous findings, our results implicate Vav1 as a relevant intracellular mediator in both NK cell cytotoxicity and CD33 inhibitory mechanism of NKG2D/DAP10-mediated lysis. Since NK cell-expressed NKG2D mediates crosstalk and regulation on other immune cells [19, 41], our data point out towards a probable function of CD33 on self-tolerance mediated by human activated NK cells.

In conclusion, this work provides evidence for the first time that CD33 functions as an inhibitory receptor preferentially for the NKG2D/DAP10 pathway, with implications in the regulation of ongoing immune responses against tumor and infected cells, in protecting self-cells and probably in avoiding autoimmunity. The biased nature of the inhibitory effect of CD33 on receptors coupled to DAP10 or SAP adaptor proteins described here provides new insights that could help to study the role of CD33 in diseases such as Alzheimer's disease.

## Data Availability

The data used to support the findings of this study are included within the article.

## Supplementary Materials

Supplementary Materials Supplementary Table 1: NKL/P815 conjugation assays were carried out by using green-labeled NKL with 400 nM calcein acetoxymethyl ester, (Ca-AM) (Molecular Probes, Eugene, OR, USA) and red-labeled P815 cells with 235 *μ*M hydroethidine (HE) (Molecular Probes) at a 1 : 2 E/T ratio and room temperature for 20 min, under the same experimental conditions used in the killing assay. Percentage of conjugates was determined by flow cytometry according to [7]. Supplementary Figure S1: activating and inhibitory receptor expression on NKL cells. High-cytotoxicity NKL cells (hc-NKL) were phenotyped by flow cytometry. Filled histograms represent isotype control whereas open histograms represent surface receptor-stained cells. Supplementary Figure S2: dose response effect of noninhibitory mAb anti-CD25 and anti-HLA-E versus inhibitory anti-CD33 mAb on the redirected lysis experiments. Anti-CD25 (clone MAR93, IgG1) and anti-HLA-E (clone 3D12, IgG1) were compared. WM53 anti-CD33 mAb were able to inhibit the lysis mediated by anti-NKG2D (0.25 *μ*g/mL) at concentrations as low as 0.075 *μ*g/mL. Supplementary Figure S3: CD33 does not inhibit cytotoxicity on quiescent primary NK cells. Redirected cytotoxic activity of purified (95%) quiescent NK cells against P815 cells was tested in the presence of different mAb against CD16, NKp46, NKG2D, or 2B4 activating receptors as described for NKL cells. The figure shows that anti-CD33 (WM53) mAb did not inhibit any activating receptor-induced killing. A representative assay is shown out of three independent experiments with similar results.

## References

[1] Varki A., Angata T. (2006). Siglecs-the major subfamily of I-type lectins. *Glycobiology*.

[2] Pillai S., Netravali I. A., Cariappa A., Mattoo H. (2012). Siglecs and immune regulation. *Annual Review of Immunology*.

[3] Macauley M. S., Crocker P. R., Paulson J. C. (2014). Siglec-mediated regulation of immune cell function in disease. *Nature Reviews Immunology*.

[4] Paul S. P., Taylor L. S., Stansbury E. K., McVicar D. W. (2000). Myeloid specific human CD33 is an inhibitory receptor with differential ITIM function in recruiting the phosphatases SHP-1 and SHP-2. *Blood*.

[5] Bradshaw E. M., Chibnik L. B., Keenan B. T. (2013). *CD33* Alzheimer’s disease locus: altered monocyte function and amyloid biology. *Nature Neuroscience*.

[6] Son M., Diamond B., Volpe B. T., Aranow C. B., Mackay M. C., Santiago-Schwarz F. (2017). Evidence for C1q-mediated crosslinking of CD33/LAIR-1 inhibitory immunoreceptors and biological control of CD33/LAIR-1 expression. *Scientific Reports*.

[7] Hernández-Caselles T., Martínez-Esparza M., Pérez-Oliva A. B. (2006). A study of CD33 (SIGLEC-3) antigen expression and function on activated human T and NK cells: two isoforms of CD33 are generated by alternative splicing. *Journal of Leukocyte Biology*.

[8] Pérez-Oliva A. B., Martínez-Esparza M., Vicente-Fernández J. J., Miguel R. C.-S., García-Peñarrubia P., Hernández-Caselles T. (2011). Epitope mapping, expression and post-translational modifications of two isoforms of CD33 (CD33M and CD33m) on lymphoid and myeloid human cells. *Glycobiology*.

[9] Handgretinger R., Schäfer H. J., Baur F. (1993). Expression of an early myelopoietic antigen (CD33) on a subset of human umbilical cord blood-derived natural killer cells. *Immunology Letters*.

[10] Dworzak M. N., Fritsch G., Fröschl G., Printz D., Gadner H. (1998). Four-color flow cytometric investigation of terminal deoxynucleotidyl transferase–positive lymphoid precursors in pediatric bone marrow: CD79a expression precedes CD19 in early B-cell ontogeny. *Blood*.

[11] Eissens D. N., Spanholtz J., van der Meer A. (2012). Defining early human NK cell developmental stages in primary and secondary lymphoid tissues. *PLoS One*.

[12] Kloess S., Ede Valverde da Silva A., Oberschmidt O. (2017). Triplebody mediates increased anti-leukemic reactivity of IL-2 activated donor natural killer (NK) cells and impairs viability of their CD33-expressing NK subset. *Frontiers in Immunology*.

[13] Walter R. B., Raden B. W., Zeng R., Häusermann P., Bernstein I. D., Cooper J. A. (2008). ITIM-dependent endocytosis of CD33-related siglecs: role of intracellular domain, tyrosine phosphorylation, and the tyrosine phosphatases, Shp1 and Shp2. *Journal of Leukocyte Biology*.

[14] Gonzalez Y., Herrera M. T., Soldevila G. (2012). High glucose concentrations induce TNF-*α* production through the down-regulation of CD33 in primary human monocytes. *BMC Immunology*.

[15] Balmer M. L., Trüeb B., Zhuang L., Slack E., Beltraminelli H., Villiger P. M. (2013). Splicing defect of CD33 and inflammatory syndrome associated with occult bacterial infection. *Journal of Allergy and Clinical Immunology*.

[16] Gasser S., Raulet D. H. (2006). Activation and self-tolerance of natural killer cells. *Immunological Reviews*.

[17] Lanier L. L. (2008). Up on the tightrope: natural killer cell activation and inhibition. *Nature Immunology*.

[18] Upshaw J. L., Arneson L. N., Schoon R. A., Dick C. J., Billadeau D. D., Leibson P. J. (2006). NKG2D-mediated signaling requires a DAP10-bound Grb2-Vav1 intermediate and phosphatidylinositol-3-kinase in human natural killer cells. *Nature Immunology*.

[19] Stojanovic A., Correia M. P., Cerwenka A. (2018). The NKG2D/NKG2DL axis in the crosstalk between lymphoid and myeloid cells in health and disease. *Frontiers in Immunology*.

[20] Latour S., Gish G., Helgason C. D., Humphries R. K., Pawson T., Veillette A. (2001). Regulation of SLAM-mediated signal transduction by SAP, the X-linked lymphoproliferative gene product. *Nature Immunology*.

[21] Chen R., Relouzat F., Roncagalli R. (2004). Molecular dissection of 2B4 signaling: implications for signal transduction by SLAM-related receptors. *Molecular and Cellular Biology*.

[22] Corral-San Miguel R., Hernández-Caselles T., Ruiz Alcaraz A. J., Martínez-Esparza M., García-Peñarrubia P. (2014). MHC-I molecules selectively inhibit cell-mediated cytotoxicity triggered by ITAM-coupled activating receptors and 2B4. *PLoS One*.

[23] Sgroi D., Varki A., Braesch-Andersen S., Stamenkovic I. (1993). CD22, a B cell-specific immunoglobulin superfamily member, is a sialic acid-binding lectin. *The Journal of Biological Chemistry*.

[24] Mosmann T. (1983). Rapid colorimetric assay for cellular growth and survival: application to proliferation and cytotoxicity assays. *Journal of Immunological Methods*.

[25] Colonna M., Navarro F., Bellón T. (1997). A common inhibitory receptor for major histocompatibility complex class I molecules on human lymphoid and myelomonocytic cells. *The Journal of Experimental Medicine*.

[26] André P., Castriconi R., Espéli M. (2004). Comparative analysis of human NK cell activation induced by NKG2D and natural cytotoxicity receptors. *European Journal of Immunology*.

[27] Moebius J. M., Widera D., Schmitz J., Kaltschmidt C., Piechaczek C. (2007). Impact of polysialylated CD56 on natural killer cell cytotoxicity. *BMC Immunology*.

[28] Kondadasula S. V., Roda J. M., Parihar R. (2008). Colocalization of the IL-12 receptor and Fc*γ*RIIIa to natural killer cell lipid rafts leads to activation of ERK and enhanced production of interferon-*γ*. *Blood*.

[29] López-Lago M., Lee H., Cruz C., Movilla N., Bustelo X. R. (2000). Tyrosine phosphorylation mediates both activation and downmodulation of the biological activity of Vav. *Molecular and Cellular Biology*.

[30] Sharma R., Das A. (2014). Organ-specific phenotypic and functional features of NK cells in humans. *Immunologic Research*.

[31] Caligiuri M. A., Zmuidzinas A., Manley T. J., Levine H., Smith K. A., Ritz J. (1990). Functional consequences of interleukin 2 receptor expression on resting human lymphocytes: identification of a novel natural killer cell subset with high affinity receptors. *Journal of Experimental Medicine*.

[32] Dalbeth N., Callan M. F. C. (2002). A subset of natural killer cells is greatly expanded within inflamed joints. *Arthritis & Rheumatism*.

[33] Beziat V., Duffy D., Quoc S. N. (2011). CD56^bright^CD16^+^ NK Cells: a functional intermediate stage of NK cell differentiation. *The Journal of Immunology*.

[34] Fujisaki H., Kakuda H., Shimasaki N. (2009). Expansion of highly cytotoxic human natural killer cells for cancer cell therapy. *Cancer Research*.

[35] Cheng M., Chen Y., Xiao W., Sun R., Tian Z. (2013). NK cell-based immunotherapy for malignant diseases. *Cellular & Molecular Immunology*.

[36] Nicoll G., Avril T., Lock K., Furukawa K., Bovin N., Crocker P. . R. (2003). Ganglioside GD3 expression on target cells can modulate NK cell cytotoxicity via siglec-7-dependent and -independent mechanisms. *European Journal of Immunology*.

[37] Cooper M. A., Fehniger T. A., Caligiuri M. A. (2001). The biology of human natural killer-cell subsets. *Trends in Immunology*.

[38] Stebbins C. C., Watzl C., Billadeau D. D., Leibson P. J., Burshtyn D. N., Long E. O. (2003). Vav1 dephosphorylation by the tyrosine phosphatase SHP-1 as a mechanism for inhibition of cellular cytotoxicity. *Molecular and Cellular Biology*.

[39] Mesecke S., Urlaub D., Busch H., Eils R., Watzl C. (2011). Integration of activating and inhibitory receptor signaling by regulated phosphorylation of Vav1 in immune cells. *Science Signaling*.

[40] Colucci F., Rosmaraki E., Bregenholt S. (2001). Functional dichotomy in natural killer cell signaling: Vav1-dependent and -independent mechanisms. *The Journal of Experimental Medicine*.

[41] Nielsen N., Odum N., Urso B., Lanier L. L., Spee P. (2012). Cytotoxicity of CD56^bright^ NK cells towards autologous activated CD4^+^ T cells is mediated through NKG2D, LFA-1 and TRAIL and dampened via CD94/NKG2A. *PLoS One*.

