# Supplementary material for: CD33 (Siglec-3) Inhibitory Function: Role in the NKG2D/DAP10 Activating Pathway
Source: J Immunol Res. 2019 Apr 15;2019:6032141. doi: 10.1155/2019/6032141 (PMC6501159; doi:10.1155/2019/6032141)
Supplement: Supplementary Materials — Supplementary Table 1: NKL/P815 conjugation assays were carried out by using green-labeled NKL with 400 nM calcein acetoxymethyl ester, (Ca-AM) (Molecular Probes, Eugene, OR, USA) and red-labeled P815 cells with 235 μM hydroethidine (HE) (Molecular Probes) at a 1 : 2 E/T ratio and room temperature for 20 min, under the same experimental conditions used in the killing assay. Percentage of conjugates was determined by flow cytometry according to [7]. Supplementary Figure S1: activating and inhibitory receptor expression on NKL cells. High-cytotoxicity NKL cells (hc-NKL) were phenotyped by flow cytometry. Filled histograms represent isotype control whereas open histograms represent surface receptor-stained cells. Supplementary Figure S2: dose response effect of noninhibitory mAb anti-CD25 and anti-HLA-E versus inhibitory anti-CD33 mAb on the redirected lysis experiments. Anti-CD25 (clone MAR93, IgG1) and anti-HLA-E (clone 3D12, IgG1) were compared. WM53 anti-CD33 mAb were able to inhibit the lysis mediated by anti-NKG2D (0.25 μg/mL) at concentrations as low as 0.075 μg/mL. Supplementary Figure S3: CD33 does not inhibit cytotoxicity on quiescent primary NK cells. Redirected cytotoxic activity of purified (95%) quiescent NK cells against P815 cells was tested in the presence of different mAb against CD16, NKp46, NKG2D, or 2B4 activating receptors as described for NKL cells. The figure shows that anti-CD33 (WM53) mAb did not inhibit any activating receptor-induced killing. A representative assay is shown out of three independent experiments with similar results. [file 6032141.f1.pdf]

Supplementary Figures and Table

Supplementary Table 1. Percentages of hc-NKL/P815 conjugation after co-engagement of CD33 with the activating receptors.

Inhibitory Ab	Activating receptors				
	**None**	**CD16**	**NKG2D**	**NKp46**	**2B4**
None	10.8 ± 0.8	13.7 ± 2.1	47.7 ± 2.3	11.6 ± 2.0	8.7 ± 0.9
Control IgG1	10.9 ± 1.3	12.1 ± 3.0	45.8 ± 4.1	13.5 ± 0.9	11.9 ± 0.6
CD33 (WM53)	**16.3 ± 0.1**	**24.3 ± 3.5**	**53.6 ± 0.3**	**19.8 ± 0.6**	**12.0 ± 1.6**

Supplementary Table 1.

NKL/P815 conjugation assays were carried out by using green labeled NKL with 400 nM calcein acetoxymethylester, (Ca-AM, Molecular Probes, Eugene, OR, USA) and red labeled P815 cells with 235 μM hydroethidine (HE, Molecular Probes) at 1:2 E/T ratio and room temperature for 20 min, under the same experimental conditions used in the killing assay. Percentage of conjugates was determined by flow cytometry according to [7].

Supplementary Figure S1. Activating and inhibitory receptor expression on NKL cells. High cytotoxic NKL cells (hc-NKL) were phenotyped by flow cytometry. Filled histograms represent isotype control whereas open histograms represent surface receptor stained cells.

Supplementary Figure S2. Dose-response effect of non-inhibitory mAb anti-CD25 and anti-HLA-E *versus* inhibitory anti-CD33 mAb on the redirected lysis experiments. Anti-CD25 (clone MAR93, IgG1) and anti-HLA-E (clone 3D12, IgG1) were compared.

WM53 anti-CD33 mAb were able to inhibit the lysis mediated by anti-NKG2D (0.25 μg/mL) at concentrations as low as 0.075 μg/mL.

Supplementary Figure S3. CD33 does not inhibit cytotoxicity on quiescent primary NK cells. Redirected cytotoxic activity of purified (95%) quiescent NK cells against P815 cells was tested in the presence of different mAb against CD16, NKp46, NKG2D or 2B4 activating receptors as described for NKL cells. The figure shows that anti-CD33 (WM53) mAb did not inhibit any activating receptor induced killing. A representative assay is shown out of three independent experiments with similar results.

Supplementary Figure S1

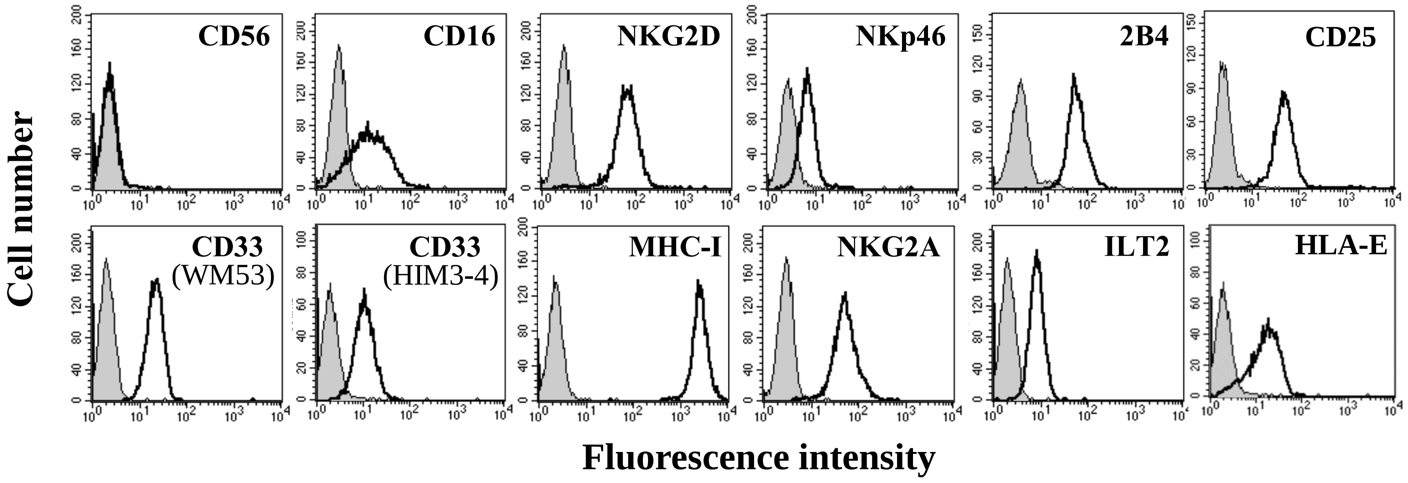

Hernández-Caselles et al. Fig. S1

Supplementary Figure S2

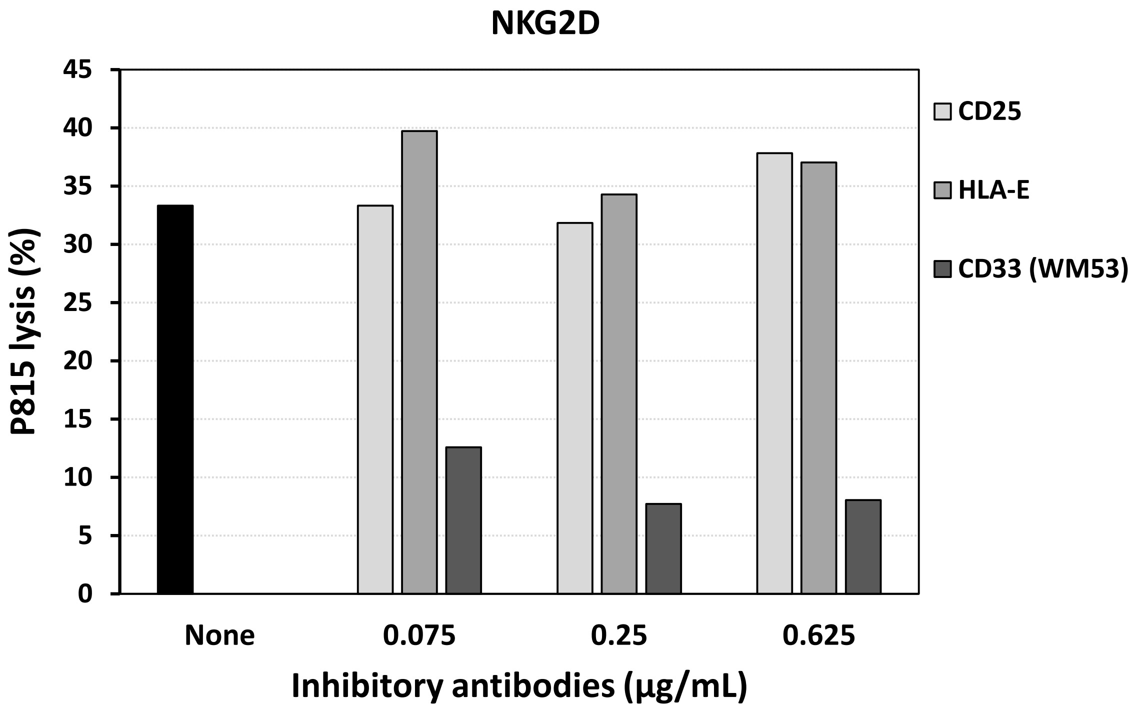

Hernández-Caselles et al. Fig. S2

Supplementary Figure S3

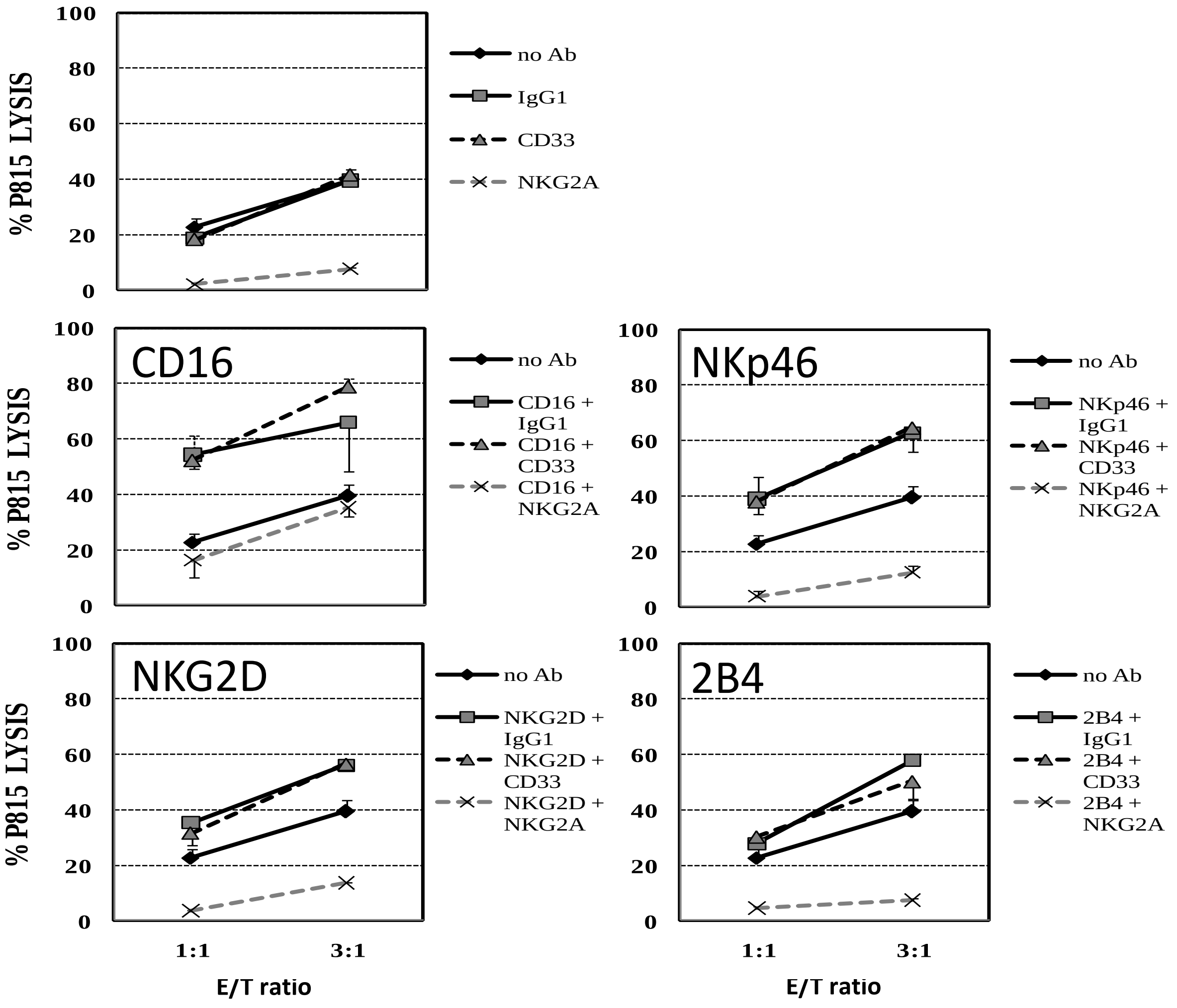

Hernández-Caselles et al. Fig. S3
